# Benefits of Hormone Therapy Estrogens Depend on Estrogen Type: 17β-Estradiol and Conjugated Equine Estrogens Have Differential Effects on Cognitive, Anxiety-Like, and Depressive-Like Behaviors and Increase Tryptophan Hydroxylase-2 mRNA Levels in Dorsal Raphe Nucleus Subregions

**DOI:** 10.3389/fnins.2016.00517

**Published:** 2016-12-08

**Authors:** Ryoko Hiroi, Giulia Weyrich, Stephanie V. Koebele, Sarah E. Mennenga, Joshua S. Talboom, Lauren T. Hewitt, Courtney N. Lavery, Perla Mendoza, Ambra Jordan, Heather A. Bimonte-Nelson

**Affiliations:** ^1^Department of Psychology, Arizona State UniversityTempe, AZ, USA; ^2^Arizona Alzheimer's ConsortiumPhoenix, AZ, USA

**Keywords:** spatial, cognition, learning and memory, serotonin, TpH2, Premarin, estrogen, mood

## Abstract

Decreased serotonin (5-HT) function is associated with numerous cognitive and affective disorders. Women are more vulnerable to these disorders and have a lower rate of 5-HT synthesis than men. Serotonergic neurons in the dorsal raphe nucleus (DRN) are a major source of 5-HT in the forebrain and play a critical role in regulation of stress-related disorders. In particular, polymorphisms of tryptophan hydroxylase-2 (TpH2, the brain-specific, rate-limiting enzyme for 5-HT biosynthesis) are implicated in cognitive and affective disorders. Administration of 17β-estradiol (E2), the most potent naturally circulating estrogen in women and rats, can have beneficial effects on cognitive, anxiety-like, and depressive-like behaviors. Moreover, E2 increases TpH2 mRNA in specific subregions of the DRN. Although conjugated equine estrogens (CEE) are a commonly prescribed estrogen component of hormone therapy in menopausal women, there is a marked gap in knowledge regarding how CEE affects these behaviors and the brain 5-HT system. Therefore, we compared the effects of CEE and E2 treatments on behavior and TpH2 mRNA. Female Sprague-Dawley rats were ovariectomized, administered either vehicle, CEE, or E2 and tested on a battery of cognitive, anxiety-like, and depressive-like behaviors. The brains of these animals were subsequently analyzed for TpH2 mRNA. Both CEE and E2 exerted beneficial behavioral effects, although efficacy depended on the distinct behavior and for cognition, on the task difficulty. Compared to CEE, E2 generally had more robust anxiolytic and antidepressant effects. E2 increased TpH2 mRNA in the caudal and mid DRN, corroborating previous findings. However, CEE increased TpH2 mRNA in the caudal and rostral, but not the mid, DRN, suggesting that distinct estrogens can have subregion-specific effects on TpH2 gene expression. We also found differential correlations between the level of TpH2 mRNA in specific DRN subregions and behavior, depending on the type of behavior. These distinct associations imply that cognition, anxiety-like, and depressive-like behaviors are modulated by unique serotonergic neurocircuitry, opening the possibility of novel avenues of targeted treatment for different types of cognitive and affective disorders.

## Introduction

Anxiety and depression are prevalent and highly comorbid with cognitive impairment (Lenze and Wetherell, 2011). In particular, women are more vulnerable to both cognitive and affective disorders than men (Lenze and Wetherell, 2011). The dynamic nature of circulating ovarian hormones, such as estrogens, may contribute to the higher rates of these disorders in women compared to men. Evidence suggests that changing or low levels of estrogens in women during peri- and post- menopause are associated with cognitive decline and affective disorders (Arpels, 1996; Lenze and Wetherell, 2011). These symptoms are often effectively treated by estrogen-including hormone therapy (HT; Best et al., 1992; Halbreich et al., 1995; Sichel et al., 1995; Gregoire et al., 1996; Bimonte-Nelson et al., 2010).

Over the years, concerted efforts have been made to elucidate the effects of HT on cognition and mood. Accumulating evidence in humans and animal models demonstrates that estrogens can have a protective role. Specifically, benefits of 17β-estradiol (E2), the most potent naturally circulating estrogen, on cognition, as well as anxiety and depression, have been realized in both clinical and preclinical settings (Caldwell and Watson, 1952; Nomikos and Spyraki, 1988; Luine and Rodriguez, 1994; Daniel et al., 1997, 2005; Bimonte and Denenberg, 1999; Fader et al., 1999; Korol and Kolo, 2002; Markham and Juraska, 2002; Estrada-Camarena et al., 2003; El-Bakri et al., 2004; Feng et al., 2004; Lund et al., 2005; Bimonte-Nelson et al., 2006; Hiroi and Neumaier, 2006; Hruska and Dohanich, 2007; Talboom et al., 2008). In women, estrogen-containing HT in the form of conjugated equine estrogens (CEE; tradename Premarin) has also been shown to reduce anxiety (Campbell and Whitehead, 1977), improve mood (Gleason et al., 2015), and enhance cognitive functions (Campbell and Whitehead, 1977; Ohkura et al., 1995).

While abundant evidence indicates beneficial effects of estrogens on various measures of cognitive function, anxiety, and mood, findings are nonetheless contradictory. The most prominent landmark findings questioning estrogens' cognitive enhancing effects were from the Women's Health Initiative Memory Study (WHIMS) funded by the National Institutes of Health. In this study, in women, CEE was found to increase the risk for probable dementia when taken concurrently with medroxyprogesterone acetate (Shumaker et al., 2003) and trended toward increasing the risk of probable dementia and mild cognitive impairment when taken alone (Shumaker et al., 2004). Following this pivotal study signifying adverse effects of CEE, the use of CEE, particularly with medroxyprogesterone acetate, dramatically decreased, while bioidentical hormones, including E2, gained popularity among women (Hersh et al., 2004; Files et al., 2011; Tsai et al., 2011). CEE is comprised of more than 10 different forms of estrogens, including estrone sulfate (a weaker form of estrogen compared to E2) as the primary form of estrogen, and only a minimal level of E2. As such, CEE may exert distinct effects on cognition and mood compared to that of E2. An effort has been made recently in a randomized, placebo-controlled clinical trial, Kronos Early Estrogen Prevention Study (KEEPS), to compare the cognitive and affective outcomes of healthy, recently menopausal women with low cardiovascular risk profiles taking either CEE- or E2- containing HT (Gleason et al., 2015). The findings from this study indicated that there were no HT effects on cognition for the hormones tested, and a differential effect of CEE vs. E2 on mood.

The rodent model has served as an invaluable tool for studying many factors and variables. Mitigating and controlling for the confounds of human research including socioeconomic status, education, age, timing and duration of HT, menopause status, and endogenous hormone interactions, rodent studies enable a systematic evaluation of the effects of HT in a controlled environment. Capitalizing on these advantages, preclinical studies have demonstrated beneficial effects of E2 using rodent models of cognition, anxiety, and depression. For instance, in rodents, E2 administration improved working and reference memory in various tests of spatial cognition (Luine and Rodriguez, 1994; Bimonte and Denenberg, 1999; Fader et al., 1999; Korol and Kolo, 2002; Markham et al., 2002; El-Bakri et al., 2004; Feng et al., 2004; Daniel et al., 2005; Bimonte-Nelson et al., 2006; Hruska and Dohanich, 2007; Talboom et al., 2008; Tsai et al., 2011). In the rodent, E2 also has been shown to decrease anxiety-like behaviors, as measured by increased center time in the open field test (OFT; Lund et al., 2005; Hiroi et al., 2006; Hiroi and Neumaier, 2006) and increased open arm time in the elevated plus maze (EPM; Nomikos and Spyraki, 1988; Lund et al., 2005). E2 also decreased immobility in the forced swim test (FST; Estrada-Camarena et al., 2003; Walf et al., 2004), suggesting antidepressant activity. Emerging evidence, however, is revealing a complex interplay of parameters governing hormonal influences on cognition (Bimonte-Nelson et al., 2010), such as menopause type (transitional or surgical; Acosta et al., 2009), time after ovarian hormone loss and age (Gibbs, 2000; Daniel et al., 2006; McLaughlin et al., 2008; Talboom et al., 2008), mode and dose of hormone administration (Diaz-Veliz et al., 1991; Díaz-Véliz et al., 1999; Frick et al., 1995; Packard and Teather, 1997; Rissanen et al., 1999; Holmes et al., 2002; Bimonte-Nelson et al., 2004, 2006; El-Bakri et al., 2004; Gresack and Frick, 2004), and whether estrogen was given alone or in conjunction with a progestogen component (Bimonte-Nelson et al., 2006; Harburger et al., 2007; Lowry et al., 2010; Chisholm and Juraska, 2012). Previous animal studies have also investigated the effects of CEE on cognition and affect. Our laboratory has demonstrated that in middle-aged Fischer-344 rats, CEE had beneficial effects on cognition, although these effects depended on multiple variables, such as the hormonal status of the rats, specific memory task used to evaluate cognition, and dosing parameters (Acosta et al., 2009, 2010; Engler-Chiurazzi et al., 2011). In addition, Frye and colleagues showed beneficial effects of CEE on cognition and anxiety-like behaviors in middle-aged Long Evans rats; however, the effects depended on the reproductive status of the rats (Frye et al., 2010). To our knowledge, whether CEE impacts depressive-like measurements in animal models has not been reported.

While underlying mechanisms for estrogen effects on cognition, anxiety, and depression are largely unknown, there are several lines of evidence that underscore the importance of the brain serotonin (5-HT) system. Notably, postmenopausal women have decreased serotonergic activity (Halbreich et al., 1995), which is associated with a higher incidence of cognitive and affective disorders (Waider et al., 2011). Estrogen treatment reverses the decreased serotonergic activity in these women (Halbreich et al., 1995) and ameliorates symptoms of cognitive and affective disorders (Campbell and Whitehead, 1977; Best et al., 1992; Ohkura et al., 1995; Arpels, 1996; Gregoire et al., 1996; Gleason et al., 2015). Studies using positron emission tomography scans have also shown a decreased rate of 5-HT synthesis in women compared to men (Okazawa et al., 2000) and in depressed compared to non-depressed patients (Rosa-Neto et al., 2004). Animal studies corroborate these findings, as baseline firing rate of 5-HT neurons in the midbrain dorsal raphe nucleus (DRN), a major source of 5-HT in the forebrain, is lower in cycling and ovariectomized (Ovx) female rats compared to that in male rats (Klink et al., 2002; Robichaud and Debonnel, 2005). Estrogen stimulates 5-HT activity, as illustrated by increased serotonergic neurotransmission during pregnancy and following exogenous E2 treatment (Klink et al., 2002; Robichaud and Debonnel, 2005). These studies together indicate that decreased serotonergic function in women contributes to vulnerability to some forms of cognitive and affective disorders, and that estrogen administration may ameliorate symptoms by increasing 5-HT neurotransmission.

Of note, tryptophan hydroxylase-2 (TpH2), a brain-specific isoform of the rate-limiting enzyme for 5-HT biosynthesis (Walther and Bader, 2003), has been implicated in the regulation of cognition, anxiety, and depression (Sun et al., 2004; Nash et al., 2005; You et al., 2005; Zhang et al., 2005). Previous studies have shown that E2 increases TpH2 mRNA in specific subregions of the DRN in Ovx rats (Hiroi et al., 2006), and that this increase is critical for ameliorating anxiety-like and depressive-like behaviors (Hiroi et al., 2006, 2011). Collectively, these studies suggest that the beneficial effects of estrogens are mediated by enhanced brain 5-HT activity, perhaps via the upregulation of TpH2 expression in specific subregions of the DRN.

Previous studies exploring the effects of CEE on the DRN 5-HT system are limited. In non-human primates, pioneering works by Bethea and colleagues have demonstrated that CEE administration increased TpH1 (the original isoform of tryptophan hydroxylase) protein levels in the DRN of Ovx adult macaques (Bethea et al., 2000; Shively et al., 2003). In light of the recent discovery of a second, brain-specific isoform, TpH2 (Walther and Bader, 2003), and demonstration of a significant relationship between the behavioral effects of E2 and the level of TpH2 mRNA in distinct subregions of the DRN (Hiroi et al., 2006, 2011; Donner and Handa, 2009), the next critical step is to examine how CEE impacts TpH2 in the subregions of the DRN. To date, however, the effects of CEE on TpH2 expression in the rodent DRN have not been examined, with a noted lack of work in varied DRN subregions. Comparing the region-specific effects of CEE vs. E2 on TpH2 may provide insight into the mechanisms underlying the behavioral effects of these estrogens.

The present study was designed to extend the previous work from our laboratory showing cognitive effects of CEE and E2 in middle-aged Fischer-344 rats (Bimonte-Nelson et al., 2006; Talboom et al., 2008; Acosta et al., 2009, 2011; Engler-Chiurazzi et al., 2011) to a younger age group and a different rat strain by evaluating the cognitive effects of these estrogens in young adult, Ovx Sprague Dawley rats. Thus far, none of the aforementioned preclinical studies have directly compared the effects of CEE and E2. In addition, to our knowledge, no previous studies have examined the effects of CEE on depressive-like behaviors or on DRN TpH2 expression in the rodent model. Therefore, in the present study, we evaluated and compared the effects of CEE and E2 administration on TpH2 mRNA expression in the DRN, and on a battery of cognitive, anxiety-like, and depressive-like behaviors in Ovx Sprague Dawley rats. The relationship between TpH2 mRNA and the different types of behaviors were also analyzed to reveal possible hormone- and subregion- specific changes in DRN TpH2 that may be important for the evaluated behaviors.

## Materials and methods

### Animals

A total of 25 female Sprague Dawley rats purchased from Charles River Laboratories (Wilmington, Massachusetts) were used for this study. Animals were pair housed, had access to food and water *ad libitum*, and were maintained in a temperature and humidity controlled environment with a 12-h light/dark cycle. Animals were 4 months old at maze initiation. All procedures were approved by the Institutional Animal Care and Use Committee and adhered to the National Institutes of Health guidelines.

### Ovariectomy and hormone manipulations

All animals underwent Ovx surgeries. Animals were anesthetized with isoflurane (Baxter HealthCare, Deerfield, IL) and bilateral incisions were made from the dorsal aspect. Ovaries and tips of uterine horns were ligatured and removed, and muscle and skin were sutured. Rats received Rimadyl (5 mg/mL/kg) for pain and saline (2 mL) to prevent dehydration. Twenty-five days following Ovx, rats were randomly assigned to one of the following treatment groups: Vehicle (sesame oil, *n* = 9), CEE (30 μg CEE in sesame oil, *n* = 8), or E2 (3 μg E2 in sesame oil, *n* = 8). One subcutaneous injection (0.1 mL volume) was given for 2 consecutive days, followed by 2 days off, and this pattern was repeated throughout the study until animals were sacrificed. The doses of CEE and E2 were based on CEE and E2 doses previously shown to enhance performance on spatial tasks (Talboom et al., 2008; Acosta et al., 2009, 2011; Engler-Chiurazzi et al., 2011). The dose of CEE used in the current study was also based on the daily 0.625 mg CEE dose commonly taken by women, and used in the WHIMS. Given the average female body weight of 70 kg (www.halls.md), we calculated 0.00893 mg CEE/kg body weight. Based on this dose, in a previous study, we utilized a range of 10–30 μg daily administration of powder, which was 10% CEE (i.e., 1 μg CEE in a 10 μg dose and 3 μg CEE in a 30 μg dose) to evaluate cognitive effects in female rats (Acosta et al., 2009; Engler-Chiurazzi et al., 2011). The highest dose of 30 μg powder (i.e., 3 μg of CEE specifically) was chosen for this study, as this dose showed the most beneficial cognitive profile in the prior work.

The treatment regimen of 2 days on and 2 days off was chosen based on previous reports showing cognitive effects of CEE in middle-aged Ovx Fischer-344 rats (Acosta et al., 2009; Engler-Chiurazzi et al., 2011). We chose this intermittent, pulsatile regimen over a continuous, tonic regimen as our laboratory found cognitive enhancements using an identical intermittent regimen of CEE treatment (Acosta et al., 2009), while a tonic CEE treatment via an osmotic pump impaired cognition (Engler-Chiurazzi et al., 2011). This temporal regimen of E2 injection also has been shown to alter hippocampal plasticity and memory (Woolley and McEwen, 1993; Korol and Kolo, 2002; McLaughlin et al., 2008). The present study was designed to extend these prior findings to a younger age group and a different rat strain, by evaluating cognition in young adult, Ovx Sprague Dawley rats. In addition, we sought to compare the cognitive effects of CEE to that of E2. Finally, we also expanded our study design to include outcome measures evaluating anxiety-like and depressive-like behaviors as well as the level of DRN TpH2 gene expression.

### Uterine weights

To confirm Ovx and hormone treatment, uterine tissues were collected at sacrifice. The uterus was cut between the cervix and the ligature remaining from Ovx, and was trimmed of all visible fat. It was immediately weighed to obtain wet weight.

### Behavioral testing

**Figure 1:**
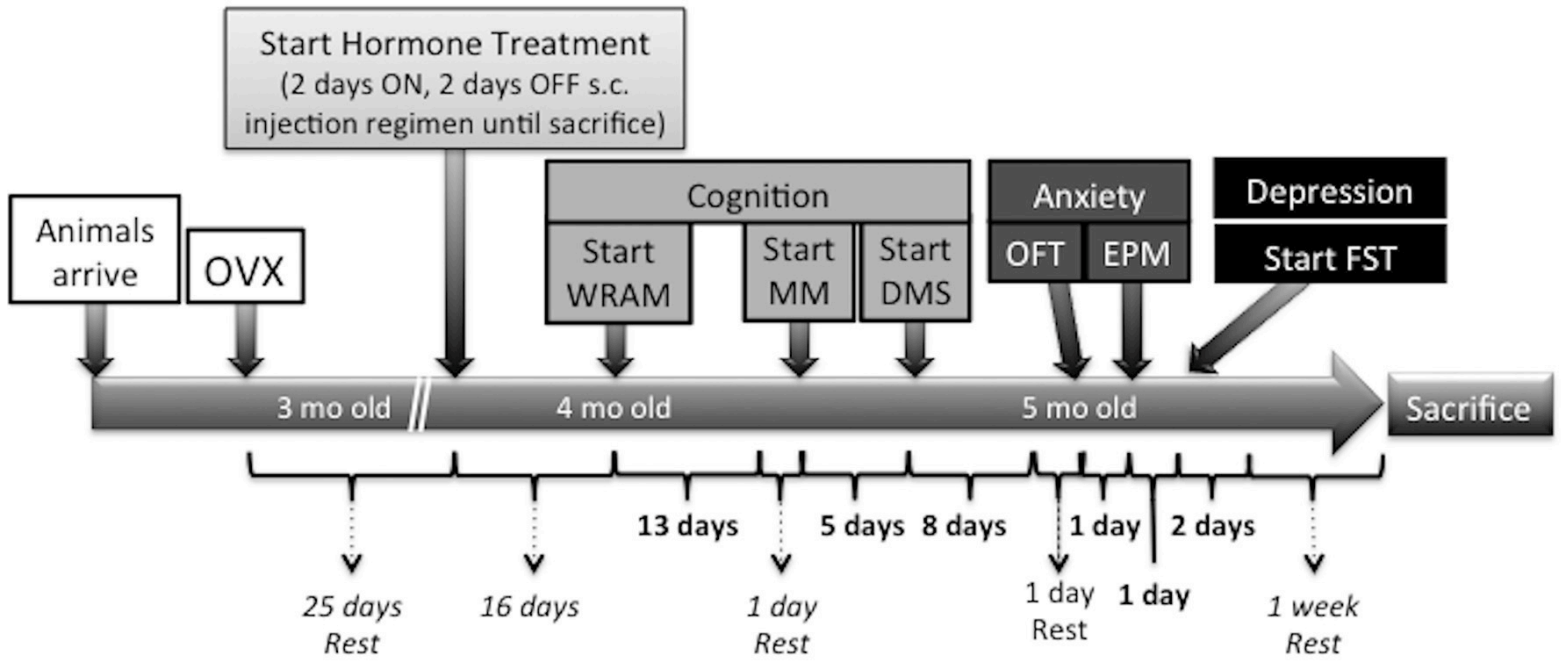
**Time course of surgeries, hormone administration, behavioral testing, and sacrifice in the present study**. Twenty-five days following Ovx, rats were randomly assigned to one of the following treatment groups: Vehicle (sesame oil, *n* = 9), CEE (30 μg CEE in sesame oil, *n* = 8), or E2 (3 μg E2 in sesame oil, *n* = 8). One subcutaneous injection (0.1 mL volume) was given for 2 consecutive days, followed by 2 days off, and this pattern was repeated throughout the study until animals were sacrificed. Sixteen days following the initiation of hormone treatment (41 days after Ovx), a series of behavioral tests commenced in the following order: water radial arm maze (WRAM), Morris maze (MM), delayed-matched-to-sample (DMS), open field test (OFT), elevated plus maze (EPM), and forced swim test (FST). One week of rest prior to sacrifice was given to all animals following the last behavioral testing day to minimize any sub-acute stress effects on gene expression. Animals were sacrificed by decapitation and brains were rapidly removed, flash frozen in 2-methylbutane at −30°C, and stored at −80°C until processed for TpH2 mRNA analysis.

Sixteen days following the initiation of hormone treatment (41 days after Ovx), a series of behavioral tests commenced in the following order: water radial arm maze (WRAM), Morris maze (MM), delayed-match-to-sample (DMS), OFT, EPM, and FST. Figure 1 depicts the timeline of the Ovx surgery, hormone treatment, and behavioral testing.

#### Water radial-arm maze

After 16 days of hormone treatment, the eight-arm WRAM was used to evaluate spatial working and reference memory (Hyde et al., 1996, 1998; Bimonte and Denenberg, 1999). Working memory is information requiring updating that is pertinent for a short time, while reference memory is information that remains constant over time (Jarrard et al., 1984; Jones, 2002; Bimonte-Nelson, 2015). The WRAM contained hidden platforms at the ends of four of the eight arms. Each animal received 1 session per day for 12 days, with platform locations remaining constant across all sessions for each animal. A session consisted of 4 trials; in a trial, an animal had 3 min to locate a platform, and once found, the animal remained on it for 15 sec. The just-found platform was removed from the maze while the animal was placed in a heated cage for a 30 sec inter-trial interval (ITI). This procedure was repeated until all remaining platforms were found, resulting in 4 trials per day. Because the number of platformed-arms decreased by 1 on each subsequent trial, the working memory load increased as trials progressed within a session.

Errors were quantified using the orthogonal measures of working and reference memory errors. First and repeated entries into arms that contained a platform that had been removed during the session were considered working memory correct errors (WMC). First entries into arms that never contained a platform were scored as reference memory errors (RM). Repeat entries into reference memory arms were scored as working memory incorrect errors (WMI).

Since animals had no prior experience with the maze on the first day, Day 1 was considered a training session and was excluded from analysis of the testing sessions, which were Days 2–12. Testing days were blocked into the acquisition phase (Days 2–7) and the asymptotic phase (Days 8–12), and each type of error was analyzed separately. Similar blocking methods have been used for WRAM analyses evaluating ovarian hormones (Bimonte et al., 2000; Bimonte-Nelson et al., 2003a,b). On Day 13, a 4-h delay was given between trials 2 and 3.

#### Morris maze

One day following the end of WRAM testing, the MM was used to test spatial reference memory. The MM was a round water-filled container (188 cm diameter) with a hidden platform remaining in a fixed location throughout testing. Each animal received 1 session per day for 5 days, with 4 trials per day. In a trial, an animal had 60 sec to locate the platform after being placed in one of the four alternating start locations: the north, south, east, or west wall of the maze. After finding the platform, the animal remained on the platform for 15 sec and then was placed into a heated cage until the next trial, with an approximate ITI of 10 min. This procedure was repeated for the remaining trials. A video camera was connected to a tracking system (Ethovision, Noldus Instruments, Wageningen, The Netherlands), which analyzed the path of each rat. Swim distance (cm) from the start location to the platform was the dependent variable.

On the last day, after completion of all trials, a probe trial was given to test whether the animals spatially localized the platform location. In the probe trial, animals were allowed to swim for 60 sec in the MM without the platform. Percent swim distance in the previously platformed (target) quadrant was compared to percent swim distance in the diagonally opposite quadrant, with the tenet that animals that had learned the spatial location of the platform should have the greatest percent of total swim distance in the target compared to the opposite quadrant.

#### Delayed-match-to-sample

One day following the end of MM testing, the win-stay DMS place learning task was used to assess spatial working memory as well as short-term memory retention (i.e., recent memory). The DMS task had a platform hidden at the end of one of the four arms. The platform location was the same within a day, but changed across days. Each animal received 1 session per day for 7 consecutive days, with 6 trials per daily session. In a trial, rats had 90 sec to locate a platform; once found, rats remained on the platform for 15 sec before being placed into a heated cage for a 30 sec ITI. This procedure was repeated for the remaining trials, with the start arm varied for each trial. Trial 1 was considered the information trial, as it informed the animal where the platform was for that session. Trial 2 was considered the working memory test trial, and trials 3–6 were considered recent memory test trials (Frick et al., 1995). Entry into any non-platformed arm was scored as an error, and the total number of errors were analyzed for each trial.

Testing days were blocked into the acquisition phase (Days 1–3) and the asymptotic phase (Days 4–7). On Day 8, animals were given a 6-h delay between the information trial (trial 1) and the working memory test trial to evaluate delayed memory retention.

#### Open field test

After 1 day of rest following the DMS, the OFT was performed to assess anxiety-like behaviors. Two open field arenas, constructed of black acrylic (100 × 100 × 40 cm), were located in adjacent rooms with identical dimensions. The rooms were dark and illuminated by a light bulb positioned over the center of the chamber, illuminating the center of the open field arena at 70–80 lux. Animals were acclimated for at least 30 min in a quiet hallway adjacent to the testing rooms. Animals were then placed in the open field arena at the drop off location (near the middle of the east wall) facing the center of the arena, and allowed to roam freely for 10 min. The behavior was videotaped for later analysis by an experimenter blind to the treatment groups. For analysis, the arena was divided into 25 equally-sized squares by lines created on a behavior tracking software, Ethovision (Noldus Instruments, Leesburg, VA). The following parameters were analyzed: time spent (sec) in the center square and the corner squares, and total line crossings.

#### Elevated plus maze

One day following the OFT, the EPM was given to assess anxiety-like behaviors. The EPM consisted of four arms, 49 cm long and 10 cm wide, elevated 50 cm off the ground. Two arms were enclosed by walls 30 cm high, and the other two arms were exposed and open. There were no raised enclosing edges or barriers on the open arms. The EPM was illuminated by a light bulb positioned over the open arms, illuminating the open arms at 70–80 lux. Rats were placed at the junction of the open and closed arms and allowed to explore the maze for 10 min. Behavior in the EPM was videotaped for later analysis by an experimenter blind to the treatment groups. The amount of time spent (sec) on the open and closed arms was analyzed.

#### Forced swim test

One day following the EPM, the FST was conducted to assess depressive-like behaviors. The FST was a two-day procedure. On the first day, the rats were placed individually into a clear Plexiglas cylinder, 30 cm high and 18 cm in diameter, containing fresh water at 25°C, filled up to 20 cm. The animals were left to swim in the water for 15 min before being removed and allowed to dry under a heat lamp in the testing cage. Twenty-four hours later, the procedure was repeated, except the rats swam for 5 min. The videotapes of the second test day were analyzed by an experimenter blind to the treatment groups, and the following behaviors were scored: immobility, defined as slight movements only necessary to keep the rat's head above water; climbing, defined as rapid movement of the forelimbs that break the water surface and/or upward, vertical movement attempting to climb against the wall; swimming, defined as all other movements.

### *In situ* hybridization histochemistry

#### Tissue preparation

One week following the last behavioral testing day, animals were sacrificed by decapitation after brief exposure to isoflurane. The brains were rapidly removed, flash frozen in 2-methylbutane at −30°C, and stored at −80°C until analysis. Frozen brains were cryosectioned at −20°C; serial 20 μm-thick coronal sections containing the DRN across the anteroposterior axis were mounted on Superfrost Plus slides (Fisher Scientific Pittsburgh, Pennsylvania) and stored at −80°C until processed for *in situ* hybridization histochemistry (ISHH). Tissue sections were briefly thawed at room temperature and fixed in 4% paraformaldehyde for 5 min. After rinsing in phosphate-buffered saline (PBS), sections were treated with acetic anhydride for 10 min, dehydrated through a series of graded alcohol solutions, and air-dried.

#### Oligoprobes and ISHH

Antisense oligoprobes complementary to rat and mouse *tph2* mRNA (5′-TCC GTC CAA ATG TTG TCA GGT GGA TCC AGC CTC ACA ATG GTG GTC-3′; position 505; NM_173391) were synthesized by and purchased from Integrated DNA Technologies (Coralville, Iowa). The oligonucleotides were labeled with [35-S]dATP (Perkin Elmer, Waltham, Massachusetts) at the 3′ end using terminal deoxynucleotidyltransferase (Roche, Tucson, AZ) and purified using the QiaQuick Nucleotide Removal kit (Qiagen, Valencia, CA). Each slide was coverslipped with 110 μl of the hybridization buffer containing 0.1 M EDTA, SSC, single-stranded salmon sperm DNA, torula yeast tRNA, Denhardt's solution, formamide, dextran sulfate, and 1M DTT. Sections were incubated in a humidified chamber for 16 h at 42°C. Following hybridization, coverslips were removed and the slides were washed four times in 1X SSC buffer for 15 min at 55°C, once in 0.5X SSC buffer for 20 min at room temperature, and once again in 0.1X SSC buffer for 20 min at room temperature. Slides were then dipped in ddH_2_O and air-dried. Slides were exposed to BioMax Kodak autoradiography film in a sealed film cassette in a dark room at room temperature for 4 days.

#### Densitometry and data analysis

The hybridization signal was quantified using ImageJ software and correlated with behavior, as previously described (Hiroi et al., 2006). In brief, the most intense hybridization signal from each subregion of each brain was measured. Tissue background within the same section of each measurement was subtracted. An experimenter blind to the treatment groups quantified the sections. Data are expressed in optical density of the TpH2 autoradiographic signal in subregions of the DRN.

### Statistical analysis

Data were analyzed separately for each behavior and brain measure. For all measures, our primary interest was to compare hormone treatment effects; therefore, planned comparisons were run comparing vehicle- vs. E2- treated animals, and comparing vehicle- vs. CEE- treated animals. For WRAM, MM, and DMS, errors or distance were analyzed using a repeated measures ANOVA, with Treatment as the between subjects variable, and Day and Trial as the repeated measures within subjects variables, as appropriate for each maze. Paired *t*-tests were used for the MM probe trial analyses; for these analyses, each treatment group was analyzed separately with percent swim distance in the Target and Opposite quadrants as the within subjects variable. The effects of the WRAM delay were analyzed by comparing the errors for each memory type on Trial 3 on the last day of testing, Day 12 (baseline), to the post-delay trial (Trial 3) for the delay day, Day 13, for each treatment group using the Student's *t*-test. To determine whether the delay affected performance on the DMS task, we compared errors on the last day of testing (Day 7, baseline) to the delay day (Day 8) for the post-delay trial. For uterine weight, OFT, EPM, FST, and TpH2 gene expression, data were analyzed using the Student's *t*-test for the aforementioned planned comparisons. Specific behavioral parameters that showed significant treatment effects were selected for behavior-TpH2 gene association analyses consisting of all animals, including all treatment groups: WRAM WMI errors from days 8–12 trial 4, DMS total errors from days 1–3 trial 2, center time in the OFT, open arm time in the EPM, and immobility in the FST. The Fisher's *r*- to *z*-test was used to analyze correlations between the specific behavior and gene expression level in each subregion of the DRN.

## Results

### Uterine weights

**Figure 2:**
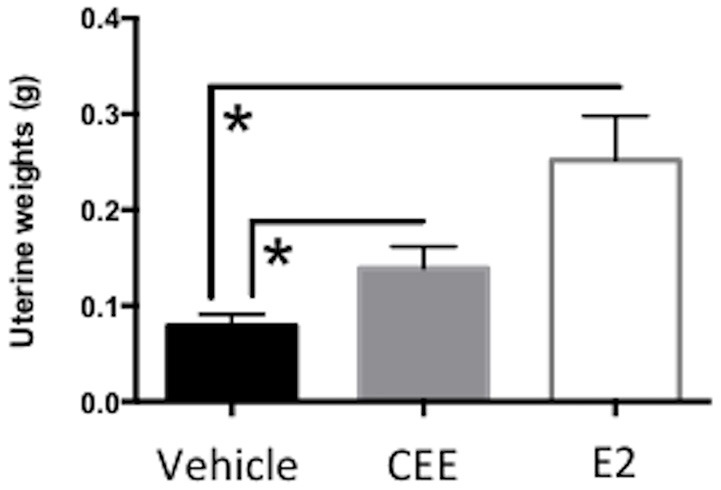
**The effects of CEE and E2 on wet uterine weights (g) at sacrifice**. To confirm Ovx and hormone treatment, uterine tissues were collected at sacrifice. The uterus was cut between the cervix and the ligature at the tips of the uterine horns remaining from Ovx, and trimmed of visible fat. The uterus was immediately weighed to obtain wet weight. Both CEE and E2 stimulated the uterus, as expected. ^*^p < 0.0001.

Both CEE- [*t*_(14)_ = 6.578, *p* < 0.0001] and E2- [*t*_(10)_ = 10.15, *p* < 0.0001] treatment increased uterine weights, with both CEE and E2 treatment groups showing greater mean weights than the Vehicle treatment group (Figure 2). These data confirmed that both CEE and E2 stimulated the uterine horns, as expected of estrogenic treatments without a progestogen component.

### Water radial-arm maze

**Figure 3:**
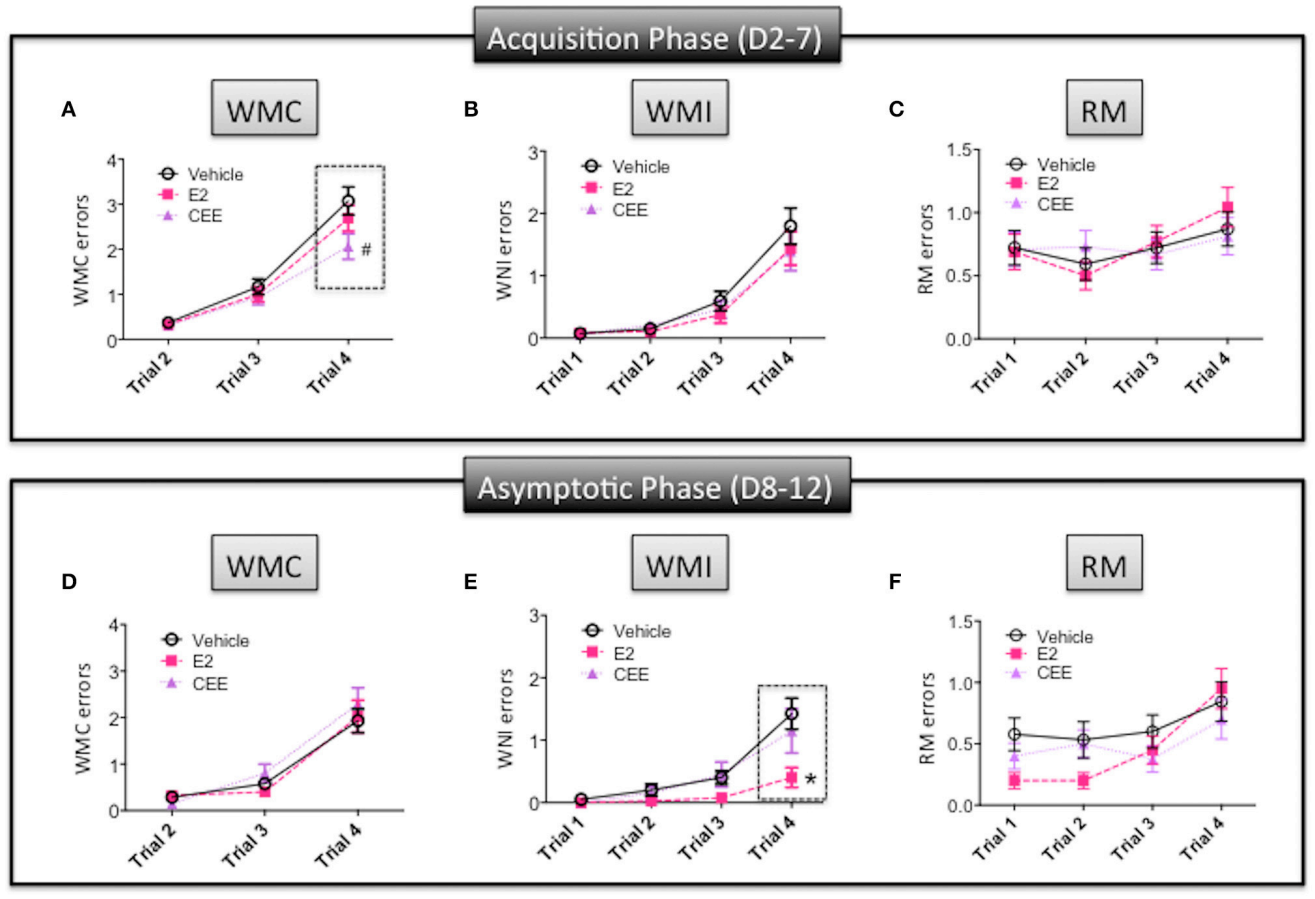
**The effects of CEE and E2 on the water radial arm maze (WRAM)**. Errors committed during the Acquisition Phase (days 2–7) for **(A)** working memory correct (WMC), **(B)** working memory incorrect (WMI), and **(C)** reference memory (RM). Errors committed during the Asymptotic Phase (days 8–12) for **(D)** WMC, **(E)** WMI, and **(F)** RM. ^*^p < 0.05, ^#^*p* < 0.10.

For the acquisition phase, there was a marginal Treatment × Trial interaction for the Vehicle vs. CEE comparison [*F*_(2, 30)_ = 2.683; *p* = 0.0848], with CEE animals making marginally fewer WMC errors than Vehicle animals on trial 4, the trial with the highest memory load [*F*_(1, 15)_ = 3.223; *p* = 0.0928; Figure 3A]. For WMI (Figure 3B) and RM (Figure 3C), neither the CEE nor E2 groups differed from the Vehicle group for the acquisition phase, nor were there significant Treatment × Trial interactions for either comparison for WMI or RM.

For the asymptotic phase, the E2 group made fewer WMI errors than the Vehicle group [*F*_(1, 14)_ = 6.409; *p* < 0.025]; however, the CEE group did not differ from the Vehicle group. For WMI, there was a significant Treatment × Trial interaction for the E2 vs. Vehicle comparison [*F*_(3, 42)_ = 4.991; *p* < 0.005], with the E2 group making fewer errors than the Vehicle group on trial 4 [*F*_(1, 15)_ = 5.765; *p* < 0.05; Figure 3E]. For WMC, neither the CEE nor E2 groups differed from the Vehicle group for the asymptotic phase, nor were there significant Treatment × Trial interactions for either comparison for WMC (Figure 3D) or RM (Figure 3F). There were no significant main effects or interactions with Treatment for RM.

**Figure 4:**
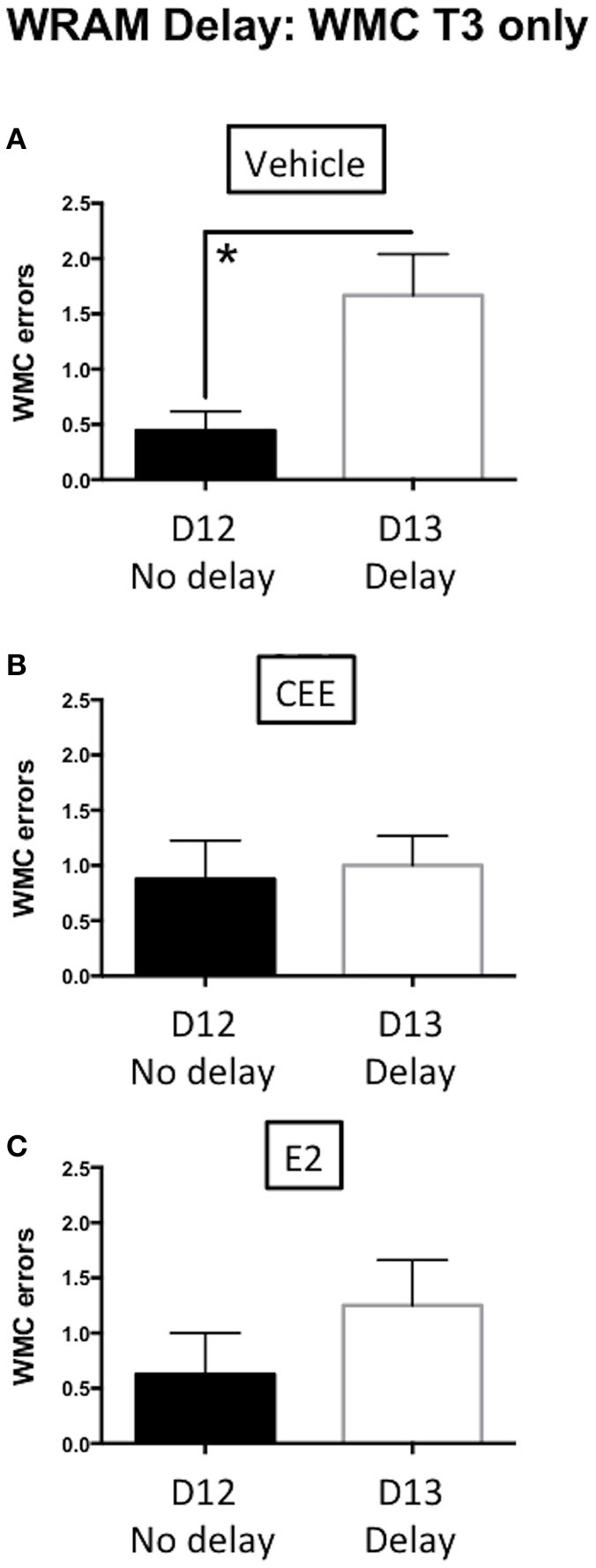
**The effects of CEE and E2 on the water radial arm maze (WRAM) delay task**. Working memory correct (WMC) errors committed by the vehicle **(A)**, CEE **(B)**, or E2 **(C)** groups during the post-delay trial (T3) following a 4-h delay between trials 2 and 3 on day 13. ^*^p < 0.01.

There was a delay-induced impairment for WMC errors for the Vehicle-treated animals [*t*_(16)_ = 2.968, *p* < 0.01] on post-delay trial 3 (Figure 4A). In contrast, there were no effects of the delay on WMC errors within each group of CEE- [*t*_(14)_ = 0.2837, *p* = 0.7808] or E2- [*t*_(14)_ = 0.1.122, *p* = 0.2808] treated animals (Figures 4B,C, respectively), suggesting that both hormone treatments prevented delay-induced impairment. There were no effects of the delay on WMI or RM errors in any of the treatment groups (data not shown).

### Morris maze

**Figure 5:**
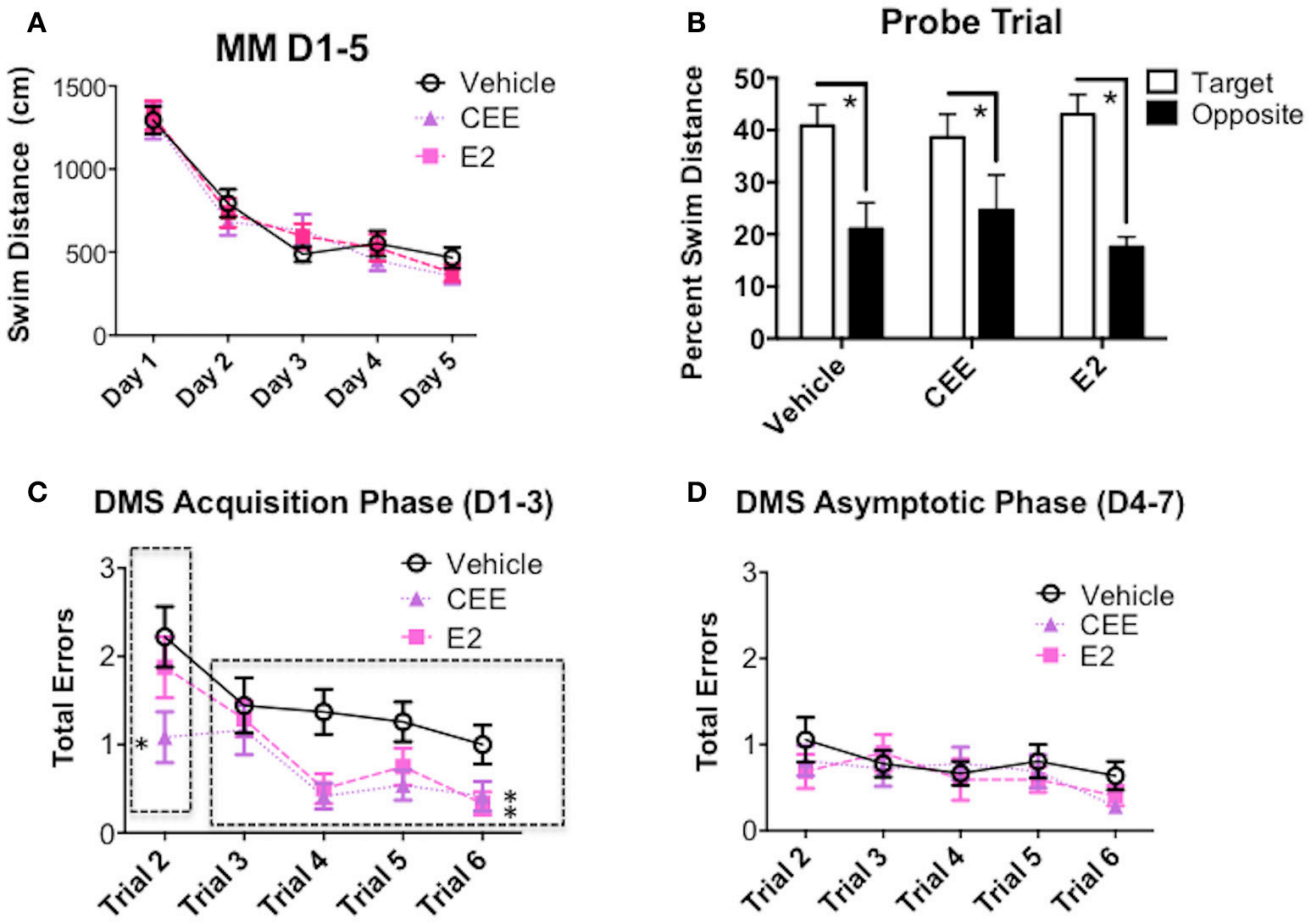
**The effects of CEE and E2 on the Morris maze (MM) and delayed-match-to-sample (DMS) behaviors. (A)** Swim distance (cm) in the MM for days 1–5. **(B)** Percent swim distance in the target vs. opposite quadrants in the MM for the probe trial assessment. Total errors committed on DMS during **(C)** the Acquisition Phase (days 1–3) and **(D)** the Asymptotic Phase (days 4–7). ^*^p < 0.05.

There were no effects of CEE or E2 treatment on MM performance across the days of testing (Figure 5A). All treatment groups spent a higher percent of their total swim distance in the target vs. opposite quadrant [Vehicle: *t*_(7)_ = 4.864, *p* < 0.0025; CEE: *t*_(6)_ = 3.423, *p* < 0.025; E2: *t*_(7)_ = 4.632, *t* < 0.0025], suggesting that all groups were able to spatially localize the platform location by the end of testing (Figure 5B).

### Delayed-matched-to-sample

During the acquisition phase of DMS testing, collapsed across all trials, the E2- [*F*_(1, 15)_ = 6.300, *p* < 0.025] and CEE- [*F*_(1, 15)_ = 9.243, *p* < 0.01] treated animals made fewer errors than the Vehicle-treated animals (Figure 5C). The CEE-treated group made fewer errors than the Vehicle-treated group for the working memory trial (trial 2) during the acquisition phase of testing [*F*_(1, 15)_ = 4.709, *p* < 0.05]. For recent memory trials (trials 3–6) both E2- [*F*_(1, 15)_ = 5.344, *p* < 0.05] and CEE- [*F*_(1, 15)_ = 5.681, *p* < 0.05] treated groups made fewer errors than the Vehicle-treated group.

During the asymptotic phase of DMS testing, neither the CEE nor E2 groups differed from the Vehicle group for any analysis (Figure 5D).

For the DMS delay trials, neither the CEE nor E2 groups differed from the Vehicle group, as all groups, regardless of treatment, made more errors on the trial after the delay compared to the same trial on the baseline day [main effect of Day for the Vehicle vs. CEE analysis: *F*_(1, 15)_ = 14.415, *p* < 0.0025; main effect of Day for the Vehicle vs. E2 analysis: *F*_(1, 15)_ = 19.881, *p* < 0.0005], with no significant Treatment × Day interactions for this trial for either analysis (data not shown).

### Open field test

**Figure 6:**
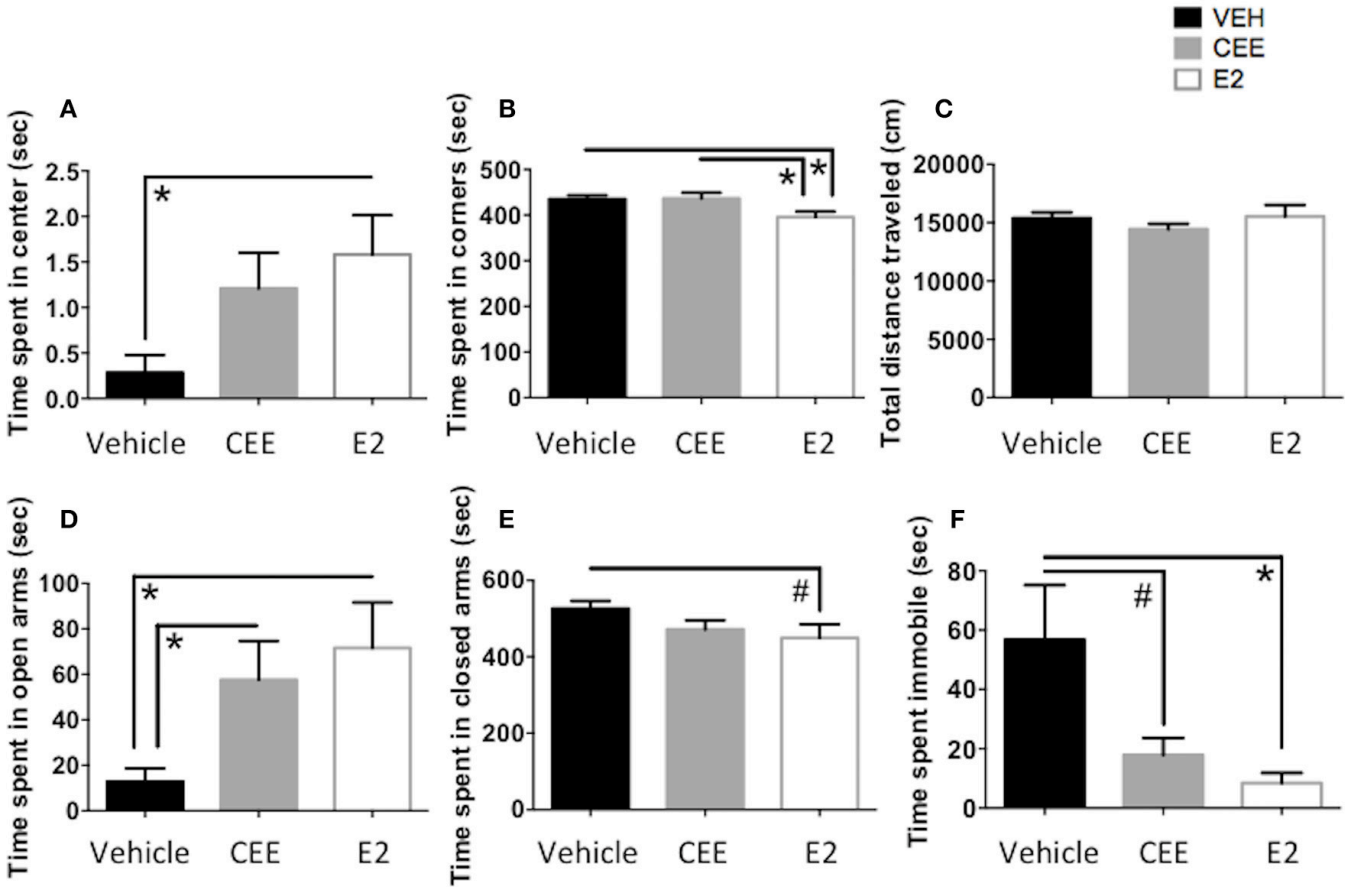
**The effects of CEE and E2 on anxiety-like and depressive-like behaviors**. Time spent in the center **(A)** and corners **(B)** and total distance traveled **(C)** in the open field test (OFT). Time spent in the open arms **(D)** and the closed arms **(E)** of the elevated plus maze (EPM). Time spent immobile **(F)** in the forced swim test (FST). ^*^p < 0.05, ^#^*p* < 0.10.

E2-treated animals spent more time in the center of the open field than Vehicle-treated animals [*t*_(14)_ = 2.666, *p* < 0.025, Figure 6A], suggesting an E2-induced decrease in anxiety-like behavior. E2-treated animals also spent less time in the corners of the open field than Vehicle-treated animals [*t*_(15)_ = 2.722, *p* < 0.025] and CEE-treated animals [*t*_(14)_ = 2.393, *p* < 0.05; Figure 6B]. CEE-treated animals also tended to spend more time in the center than Vehicle-treated animals [*t*_(14)_ = 2.123, *p* = 0.0521]. There were no effects of E2 or CEE on total distance traveled (Figure 6C), indicating that overall locomotion was not affected by the estrogen treatments. Collectively, these results suggest that E2 treatment decreased anxiety-like behaviors in the OFT, without affecting overall locomotion.

### Elevated plus maze

E2-treated animals spent more time in the open arms of the EPM than Vehicle-treated animals [*t*_(10)_ = 2.821, *p* < 0.025]; CEE-treated animals also spent more time in the open arms than the Vehicle-treated animals [*t*_(10)_ = 2.476, *p* < 0.05], suggesting that both CEE and E2 treatment are related to decreased anxiety-like behavior in the EPM (Figure 6D). Although E2-treated animals also tended to spend less time in the closed arms of the EPM than the Vehicle-treated animals, this difference was marginal [*t*_(11)_ = 1.934, *p* = 0.0792], and there were no effects of CEE on time spent in the closed arms of the EPM (Figure 6E).

### Forced swim test

E2-treated animals spent less time immobile in the FST than Vehicle-treated animals [*t*_(14)_ = 2.294, *p* < 0.05, Figure 6F]. CEE-treated animals also tended to spend less time immobile than the Vehicle-treated animals, although this difference was marginal [*t*_(14)_ = 1.811, *p* = 0.0916, Figure 6F]. There were no effects of either estrogen on time spent swimming or climbing (data not shown).

### TpH2 *in situ* hybridization histochemistry

**Figure 7:**
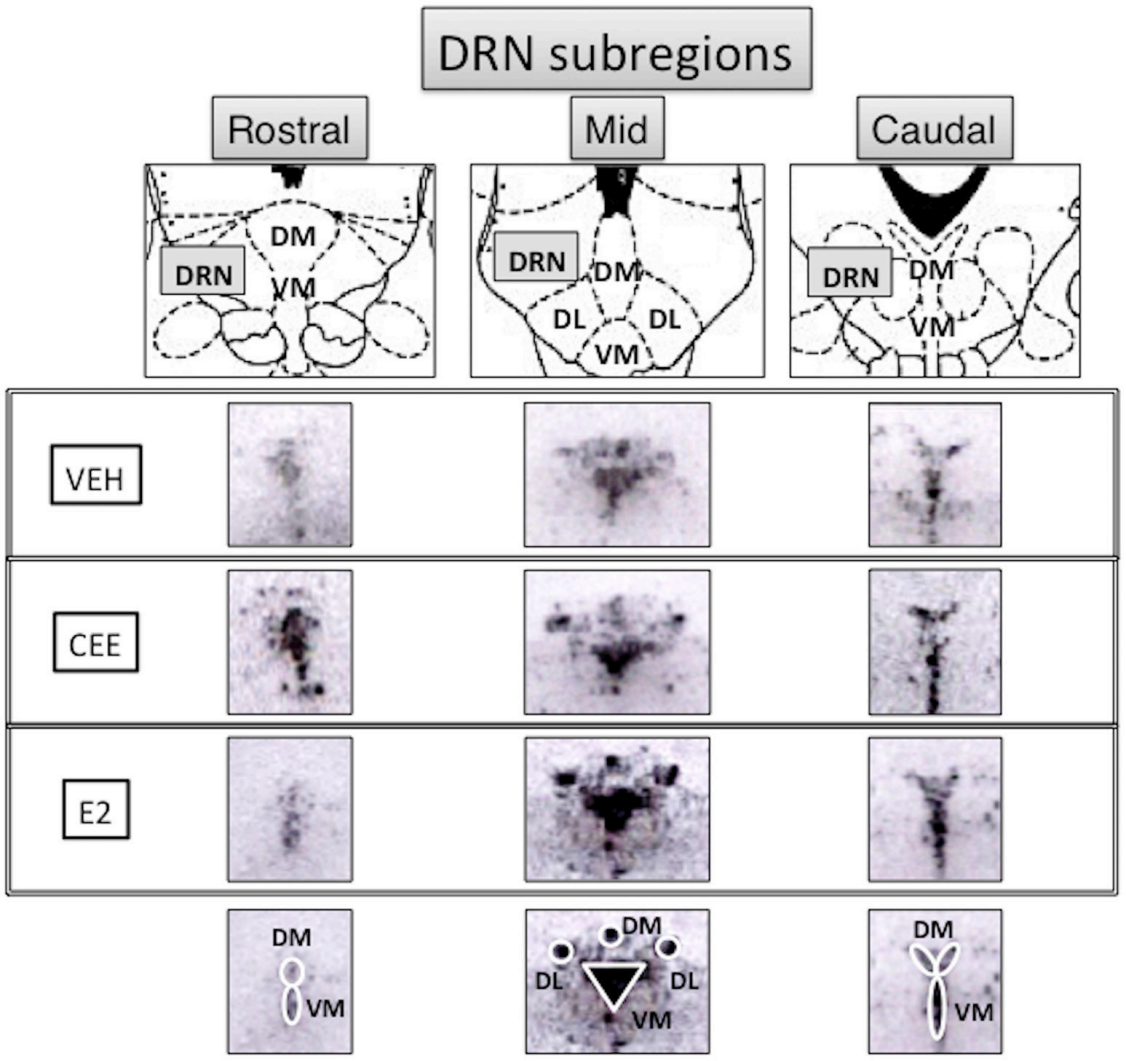
**Subregional anatomy of the raphe nuclei**. Top panels show the schematic diagrams (Paxinos and Watson, 1986) and middle panels show the representative photomicrographs of the subregions analyzed for TpH2 *in situ* hybridization signal in rostral, mid, and caudal levels of the midbrain raphe from each treatment group. Ovals and a triangle are shown on the bottom panels to illustrate the subregions analyzed for the densitometry. DRN, dorsal raphe nucleus; DM, dorsomedial; VM, ventromedial; DL, dorsolateral.

Seven DRN subregions were analyzed separately (Figure 7): the rostral DRN included the (1) dorsomedial and (2) ventromedial DRN (rDM and rVM, respectively); the mid DRN included the (3) dorsolateral, (4) dorsomedial, and (5) ventromedial DRN (mDL, mDM, mVM, respectively); the caudal DRN included the (6) dorsomedial and (7) ventromedial DRN (cDM, cVM, respectively). Two halves of the mDL and cDM subregions were averaged and used as one value for each subregion.

#### Rostral DRN

**Figure 8:**
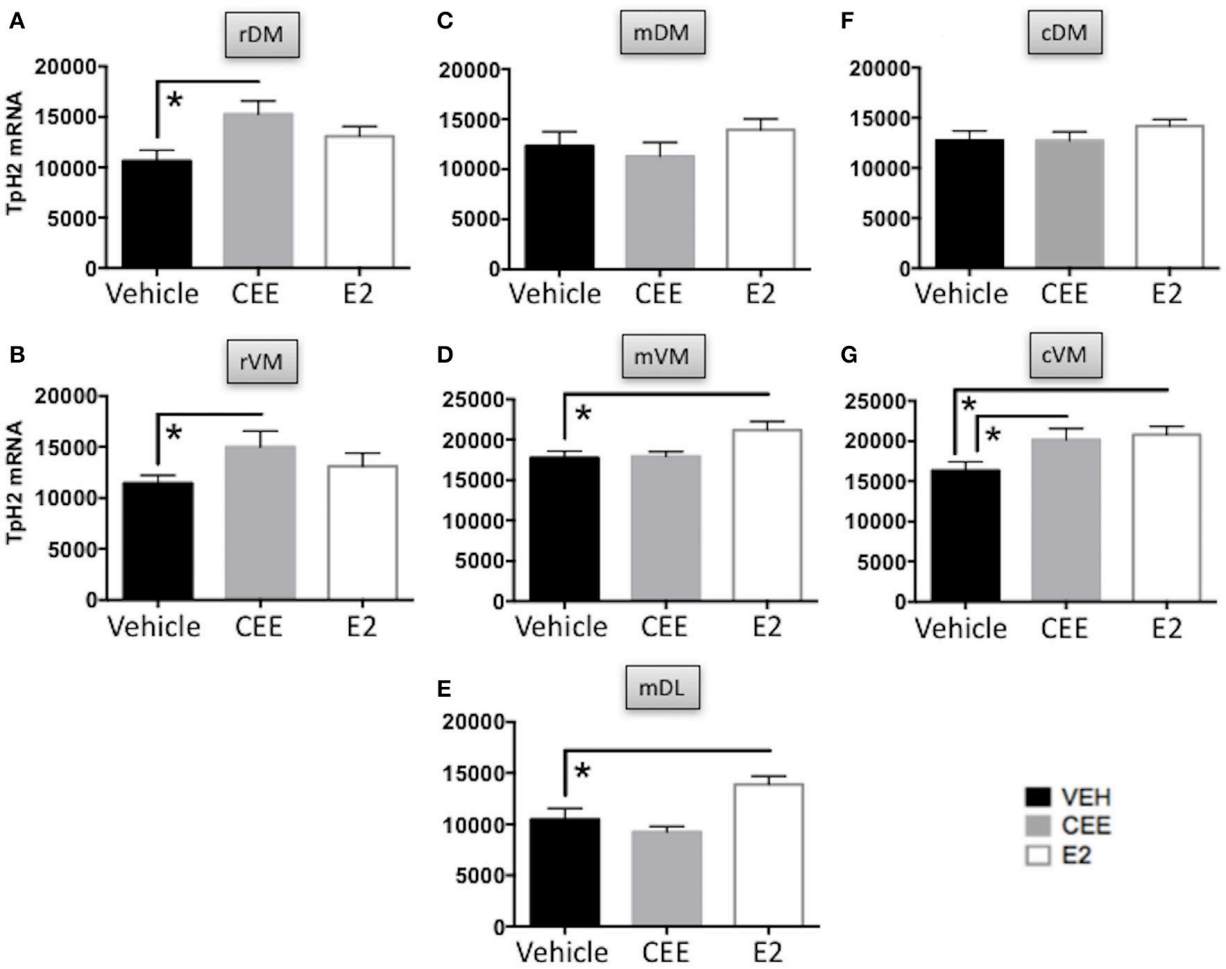
**The effects of CEE and E2 on TpH2 mRNA in the subregions of the DRN**. TpH2 mRNA *in situ* hybridization signal in the rostral **(A)** dorsomedial (rDM) and **(B)** ventromedial (rVM); medial **(C)** dorsomedial (mDM), **(D)** ventromedial (mVM), and **(E)** dorsolateral (mDL); caudal **(F)** dorsomedial (cDM), and **(G)** ventromedial (cVM) DRN. Each bar represents the mean ± SEM TpH2 optical density. ^*^p < 0.05.

CEE treatment increased the TpH2 mRNA signal in the rDM [*t*_(13)_ = 2.727, *p* < 0.025] and in the rVM [*t*_(13)_ = 2.233, *p* < 0.05], compared to Vehicle treatment (Figures 8A,B). There were no effects of E2 on TpH2 mRNA in either the rDM or rVM (Figures 8A,B).

#### Mid DRN

There were no effects of CEE or E2 on TpH2 mRNA in the mDM, compared to Vehicle treatment (Figure 8C). In contrast, E2 treatment increased TpH2 mRNA in the mVM [*t*_(15)_ = 2.598, *p* < 0.025] and in the mDL [*t*_(15)_ = 2.502, *p* < 0.025], compared to Vehicle treatment (Figures 8D,E). There were no effects of CEE on TpH2 mRNA in either the mVM or mDL (Figures 8D,E).

#### Caudal DRN

There were no effects of CEE or E2 on TpH2 mRNA in the cDM, compared to Vehicle treatment (Figure 8F). In contrast, both CEE [*t*_(13)_ = 2.205, *p* < 0.05] and E2 [*t*_(13)_ = 2.904, *p* < 0.025] increased TpH2 mRNA in the cVM, compared to Vehicle treatment (Figure 8G).

### Correlation between TpH2 gene expression and behavior

Table 1 lists the correlation matrix, highlighting significant correlations, between TpH2 mRNA levels in distinct subregions of the DRN and behavioral measures from all animals, regardless of treatment.

**Table 1:** **Correlation matrix (Pearson *r*) between behavioral measures and TpH2 mRNA in distinct subregions of the DRN**.

**Correlation coefficient (Pearson *r*)**	**TPH2 rDM**	**TPH2 rVM**	**TPH2 mDL**	**TPH2 rDM**	**TPH2 mVM**	**TPH2 cDM**	**TPH2 cVM**
WMI errors WRAM	−0.087	−0.119	−0.306	0.003	−0.281	−0.178	−0.255
Total errors DMS	−0.433^*^	−0.219	0.232	0.013	0.062	−0.025	−0.303
Center time OFT	0.412^#^	0.267	0.512^**^	0.310	0.427^*^	0.320	0.432^#^
Open arm time EPM	0.663^***^	0.136	0.432	0.530^**^	0.269	0.413^#^	0.523^*^
Immobility FST	−0.363	−0.486^*^	−0.184	−0.053	−0.141	−0.228	−0.466^*^

*** *p < 0.01*,

** *p < 0.025*,

* *p < 0.05*,

# *p < 0.1 using Fisher's r to z test. Bold values denote statistical significance of p < 0.05*.

#### Rostral DRN

**Figure 9:**
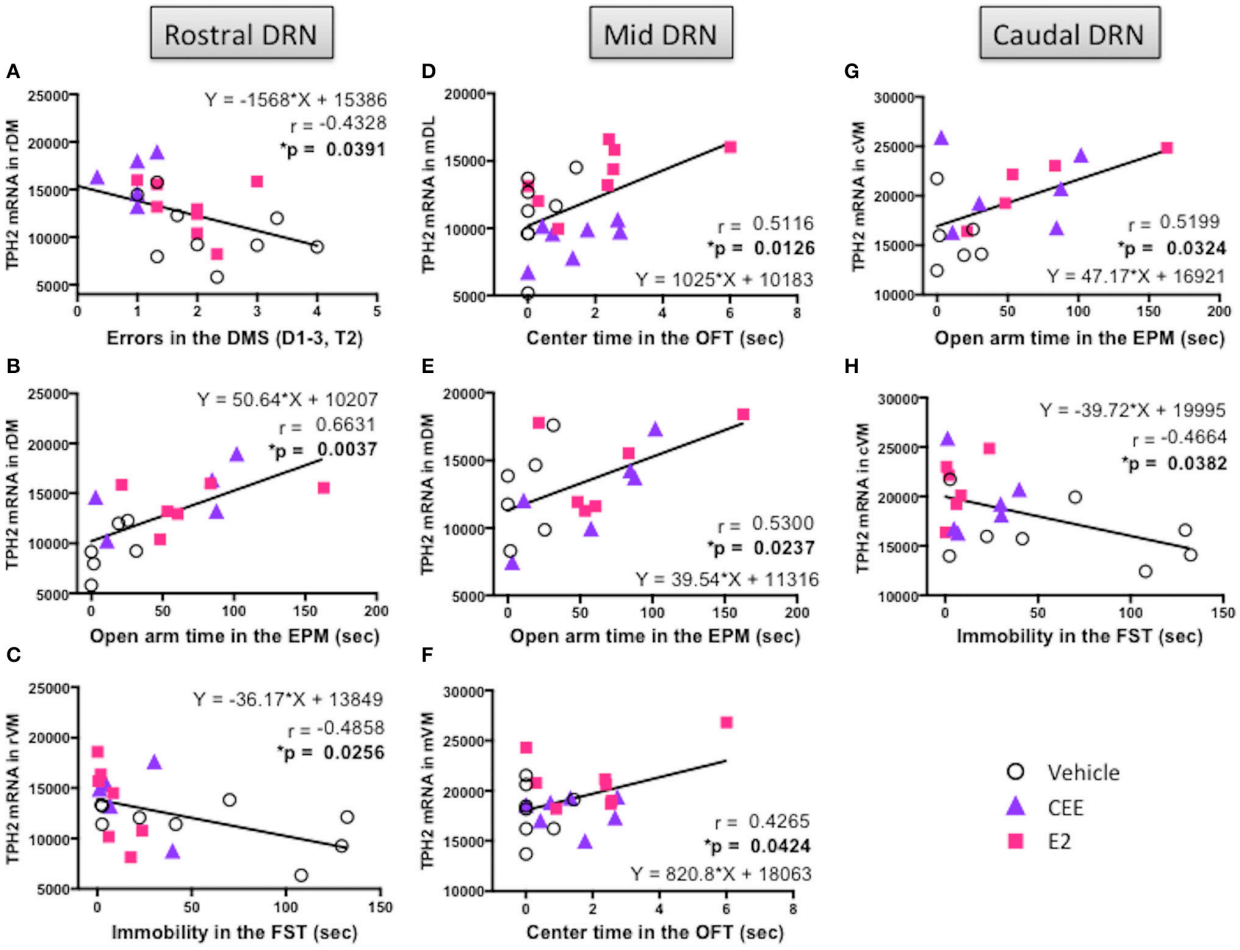
**Correlation between TpH2 mRNA in the subregions of DRN and cognitive, anxiety-like, and depressive-like behaviors**. Significant correlations using the Fisher's *r*- to *z*-test between TpH2 mRNA in the **(A)** rDM and total errors in the delayed-match-to-sample (DMS) task, **(B)** rDM and open arm time in the elevated plus maze (EPM), **(C)** rVM and immobility in the forced swim test (FST), **(D)** mDL and center time in the open field test (OFT), **(E)** mDM and open arm time in the EPM, **(F)** mVM and center time in the OFT, **(G)** cVM and open arm time in the EPM, and **(H)** cVM and immobility in the FST.

In the rDM, there was a negative correlation between TpH2 mRNA levels and total errors in the DMS [*r*_(21)_ = −0.4328, *p* < 0.05; Figure 9A], suggesting that higher levels of TpH2 in this region are associated with better performance in this task. There was also a positive correlation between TpH2 mRNA levels in the rDM and open arm time in the EPM [*r*_(15)_ = 0.6631, *p* < 0.005; Figure 9B], indicating that animals with higher levels of TpH2 in this region tend to have decreased anxiety-like behaviors in the EPM. In the rVM, there was a negative correlation between TpH2 mRNA levels and immobility in the FST [*r*_(19)_ = −0.4858, *p* < 0.05; Figure 9C], indicating that higher levels of TpH2 in this region are associated with decreased depressive-like behaviors.

#### Mid DRN

In the mDL, there was a positive correlation between TpH2 mRNA levels and center time in the OFT [*r*_(21)_ = 0.5116, *p* < 0.025; Figure 9D], suggesting that higher levels of TpH2 in this region are associated with decreased anxiety-like behaviors in the OFT. In the mVM, there was a positive correlation between TpH2 mRNA levels and center time in the OFT [*r*_(21)_ = 0.4265; *p* < 0.05; Figure 9F], suggesting that higher levels of TpH2 in this region are associated with decreased anxiety-like behaviors in the OFT. However, it is noteworthy that these two particular positive correlations may primarily result from one E2 treated animal with the highest center time (i.e., the far right data point in Figures 9D,F), as the two correlations become null when this data point is removed from analysis. Yet, this data point was not a statistical outlier and therefore was left in the analysis. Interpretation should be made with careful consideration of this caveat. We also found a positive correlation between TpH2 mRNA levels in the mDM and open arm time in the EPM [*r*_(16)_ = 0.5300; *p* < 0.025; Figure 9E], indicating that higher levels of TpH2 are associated with decreased anxiety-like behaviors in the EPM.

#### Caudal DRN

We found a positive correlation between TpH2 mRNA levels in the cVM and open arm time in the EPM [*r*_(15)_ = 0.5199, *p* < 0.05; Figure 9G], suggesting that higher levels of TpH2 in this region are associated with decreased anxiety-like behaviors in the EPM. There was also a negative correlation between TpH2 mRNA levels in the cVM and immobility in the FST [*r*_(18)_ = −0.4664, *p* < 0.05; Figure 9H], indicating that higher levels of TpH2 in this region are associated with decreased depressive-like behaviors.

## Discussion

The present study was designed to compare the effects of two commonly used estrogens in HT, CEE and E2, on a battery of cognitive, anxiety-like, and depressive-like behaviors. We also investigated the nature of the subregion-specific relationships between the brain 5-HT system and the evaluated behaviors. Although both hormone treatments generally had beneficial effects on the assessed behaviors, CEE and E2 treatments resulted in a number of hormone-specific effects with regard to the degree of the improvement, depending on the type of behavior. These differential behavioral effects may be reflected by the distinct impact of CEE vs. E2 on the brain serotonergic system, as we report subregion-specific effects on TpH2 mRNA expression in the DRN. Finally, we demonstrate here subregion-specific relationships between the DRN TpH2 mRNA and each of the evaluated behaviors, giving credence to the idea that select 5-HT output from the distinct subregions of the DRN may be involved in modulating different aspects of cognitive vs. anxiety-like vs. depressive-like behaviors.

### Beneficial cognitive effects of CEE and E2 treatments are dependent on multiple factors

The current findings add to the growing evidence that estrogens can have beneficial effects on cognition across different age groups and rat strains. For instance, in rats, the beneficial effects of estrogens have been demonstrated across the lifespan ranging from young-adult, middle-aged, to aged timeframes, as well as across various strains of rats, including Sprague Dawley (Luine et al., 1998; Sandstrom and Williams, 2001; Korol and Kolo, 2002), Purdue-Wistar (Bimonte and Denenberg, 1999), Long-Evans (Daniel et al., 1997; Markham et al., 2002; Rodgers et al., 2010), and Fischer-344 (Bimonte-Nelson et al., 2003a, 2006; Acosta et al., 2010; Engler-Chiurazzi et al., 2011).

It is important to note, however, that whether the beneficial effects of estrogens are realized may be dependent on multiple factors. First, our findings, not surprisingly, based on the state of the literature, demonstrate that the effects of estrogens are task- and memory domain- dependent. We report here that the estrogen treatments differentially enhanced performance on the WRAM, depending on the type of learning and memory. Specifically, we found that CEE, but not E2, tended to enhance working memory on the trials with the highest memory load during the acquisition phase, but not the latter, asymptotic phase of testing in the WRAM task for WMC. In contrast, E2, but not CEE, improved working memory on the trials with the highest memory load during the latter phase of testing in the WRAM task for WMI. Together, these results suggest that while cognitive benefits of CEE appeared early in the acquisition, or learning, phase of testing, E2 had an impact on working memory later in the asymptotic phase of testing. When challenged with a more demanding extended temporal retention component of the WRAM task, both CEE and E2 treatments in the present study improved memory retention across a 4-h delay. Although the present study is the first to our knowledge to report benefits of E2 administration in female rats in delayed recall of this specific task, E2 treatment has been previously shown to improve memory retention of a DMS version of the Morris water maze task after a 30- and 100- min retention interval in young adult Ovx Sprague Dawley rats (Sandstrom and Williams, 2001, 2004). Similarly, E2 also enhanced performance during 1 min and 3 h delay trials of a land version of an eight-arm radial maze (Bohacek and Daniel, 2007). Further corroborating the beneficial cognitive effects of estrogens on memory retention, our laboratory has also previously reported a post-delay protection with the same dose of CEE treatment in middle-aged Ovx Fischer-344 rats (Acosta et al., 2010; Engler-Chiurazzi et al., 2011). Delayed recall challenges the animals with a demanding task and involves retention of information over an intermediate range of time between short- and long- term memory processing. We also found that the benefits of estrogens were observed only on the trials with the highest memory load. Taken together, we speculate that the benefits of estrogens are more readily manifested when animals are challenged with a difficult task.

In contrast to our previous study showing that CEE-treated middle-aged Ovx animals made fewer reference memory errors in the latter phase of testing than those given vehicle (Acosta et al., 2010), the current study did not find an effect of CEE on reference memory during any phase of WRAM testing. It is important to note that the present study was conducted in young rats, whereas middle-aged rats were used in the previous study by Acosta et al. (2010). Given that there is a marked decline in memory around middle-age (Markowska, 1999; Talboom et al., 2008, 2014), the age difference between the two studies likely, at least in part, accounts for the different results across studies. Specifically, compared to the young adult rats evaluated in the present study, it is possible that the middle-aged animals from the previous study exhibited worse reference memory performance due to an age-related decline in baseline cognitive abilities, which interacted with a cognitive impact of estrogen. Human research corroborates this concept; for instance, the WHIMS results showed that estrogen treatment had the greatest influence in women with the lowest baseline cognitive scores (Coker et al., 2009). Another study also showed that premenopausal cognitive ability largely explained the adverse effects of menopause on cognition (Kok et al., 2006). Collectively, these studies suggest that the degree to which the cognitive effects of estrogen are manifested partially depends on baseline cognitive abilities.

In addition to memory types, task difficulty, and baseline cognitive abilities, hormone administration regimen also plays an important role in modulating the cognitive outcome of estrogens. Using an intermittent administration regimen (i.e., 2 days on and 2 days off), the present study using Sprague Dawley rats revealed beneficial effects of both CEE and E2 treatments on DMS performance during the acquisition phase. Our laboratory has previously shown similar enhancements in DMS performance using the same intermittent regimen of CEE treatment in middle-aged Ovx Fischer-344 rats (Acosta et al., 2009). On the other hand, a tonic CEE treatment via an osmotic pump impaired, rather than enhanced, performance in the same DMS task (Engler-Chiurazzi et al., 2011). Therefore, the intermittent estrogen treatment regimen may pose an advantage over the tonic regimen with respect to cognitive function as tested on the DMS task. Indeed, a previous study also showed enhanced spatial memory performance with a biweekly intermittent E2 treatment compared to tonic treatment (Bimonte-Nelson et al., 2006). Intermittent E2 treatments have been shown to enhance performance on a delayed match-to-position T-maze task in Ovx rats (Gibbs, 2000), and a spatial working memory task in Ovx rhesus monkeys (Rapp et al., 2003) as well. In agreement with the idea that intermittent administration has favorable cognitive outcomes, cognitive responsiveness to tonic E2 treatment was enhanced when primed with E2 (Markowska and Savonenko, 2002). It is also noteworthy that in a previous study, an intermittent E2 treatment did not have an effect on mortality rate, whereas a tonic E2 treatment dramatically increased mortality rate, in middle-aged Ovx rats (Bimonte-Nelson et al., 2006), suggesting an improved safety profile with the intermittent regimen over the tonic regimen. Although the underlying mechanism for this difference is unclear, evidence suggests that chronic vs. acute injections of estrogens lead to differential estrogen receptor content and dynamics, with acute injections resulting in rapid degradation followed by gradual replenishment of estrogen receptors (Kassis and Gorski, 1981; Sato et al., 1983; Rosser et al., 1993). This receptor recycling may have important consequences of E2 actions on physiology, feedback mechanisms, and ultimately, behavior. Given that the half-life of E2 is about 2 hours (Kassis and Gorski, 1981), it is possible that the intermittent regimen of 2 days on and off used in the present study would result in a receptor recycling profile impacting behavior. Future studies investigating the profile of estrogen receptor recycling status may reveal important insights into the underlying mechanism for the present reported findings.

Other important factors that impact the cognitive outcome of estrogens include duration and dose of estrogen treatment, as well as the menopause status of the animals. There is some evidence showing that a non-acute, chronic E2 treatment is required for a beneficial outcome in rats. As demonstrated by Luine et al. (1998), 12 days, but not 3 days, of E2 treatment improved choice accuracy in an eight arms baited radial-arm maze paradigm. Dose of estrogen also plays an important role. For instance, compared to the 30 μg CEE dose, lower doses of CEE (12 and 24 μg) had no protective effects on memory retention (Engler-Chiurazzi et al., 2011). Furthermore, although 30 μg CEE had beneficial effects on memory retention in Ovx rats, this retention benefit was not found in transitionally menopausal rats treated with the same dose of CEE (Acosta et al., 2010), suggesting that menopause status impacts whether protective effects are realized.

Given that both CEE and E2 can be metabolized into weaker estrogens, such as estrone, the interaction between different types of estrogens may be a critical factor in predicting the extent, and in some cases the direction, of the cognitive outcomes. To illustrate the impact of estrogen ratios on cognition, we have previously shown that the dose-dependent effects of CEE on working memory were related to the differences in resulting circulating ratios of estrone to E2 (Engler-Chiurazzi et al., 2011). Specifically, the lowest dose of CEE had no protective effects and it increased serum estrone without changing E2 levels. In contrast, the higher dose of CEE that enhanced cognitive performance resulted in increased serum levels of estrone as well as E2. In addition, we have reported that continuous estrone treatment impaired spatial memory in Ovx rats (Engler-Chiurazzi et al., 2012). These studies, collectively, imply that elevated levels of estrone in the simultaneous absence of moderate E2 levels can lead to detrimental cognitive effects. Of note, there is evidence that E2 can have detrimental cognitive effects when not metabolized to estrone. For example, we recently examined the cognitive effects of ethinyl estradiol (EE), a synthetic form of E2 used in oral contraceptives, which cannot be converted to weaker estrogens. We found that a daily dose of EE (0.3 μg) impaired multiple domains of memory in Ovx rats (Mennenga et al., 2015). Together, these studies suggest that exogenous administration of estrogens can have significant interactions with the circulating levels of its metabolites to impact behavior, depending on the ratio of distinct estrogens.

The importance of considering hormone interactions extends to other ovarian hormones and their synthetic counterparts. In particular, progestins are prescribed in women to offset the risks associated with the use of unopposed estrogens, including venous thromboembolism, stroke, endometrial hyperplasia, and endometrial cancer (Smith et al., 1975; Ziel and Finkle, 1975; Mørch et al., 2016; Swanepoel et al., 2016). Compared to estrogen alone, combined estrogen and progestin treatment can have distinct cognitive effects, depending on multiple factors (for review, please see Acosta et al., 2013). Therefore, a careful assessment of the different HT formulations, and how these exogenous hormones interact with the endogenous hormone milieu in women, may yield personalized options that have a safer physiological risk profile with improved cognitive outcomes.

In summary, the herein findings suggest that a careful consideration of parameters such as type of memory, administration regimen, menopausal status, and type of estrogens is warranted when evaluating the cognitive effects of estrogens. Over the years, there have been numerous review articles underscoring the importance of these factors (Gibbs and Gabor, 2003; Brinton, 2004; Bimonte-Nelson et al., 2010; Rocca et al., 2011; Maki, 2012; Sherwin, 2012; Acosta et al., 2013; Chisholm and Juraska, 2013; Fischer et al., 2014; Luine, 2014; Koebele and Bimonte-Nelson, 2015; McCarrey and Resnick, 2015). The present study adds to the growing consensus that clarity gained from understanding the impact of these parameters is critical for providing information to offer unique and tailored treatment options for women.

### E2 and, to a lesser extent, CEE treatments decreased anxiety-like and depressive-like behaviors

The benefits of estrogen treatments extended to anxiety-like behaviors. We showed that E2 decreased anxiety-like behaviors in the OFT and EPM, consistent with published literature (Nomikos and Spyraki, 1988; Lund et al., 2005, 2006; Hiroi et al., 2006; Hiroi and Neumaier, 2006). However, the anxiolytic effects of CEE may not be as robust as those of E2; although CEE tended to have anxiolytic effects, especially in the EPM, it did not reach statistical significance in the OFT in the current study. Previous research evaluating the effects of CEE on anxiety-like behaviors also revealed inconsistent anxiolytic effects; research has demonstrated that CEE decreased anxiety-like behaviors in the EPM, but not in the OFT, using middle-aged ovary-intact rats (Walf and Frye, 2008). Another study from the same laboratory showed that CEE decreased anxiety-like behaviors both in the OFT and EPM, although the anxiolytic effects depended on the reproductive status of the rats (Frye et al., 2010). Specifically, this study reported that the anxiolytic effects of CEE were found in rats maintaining normal reproductive status (i.e., 4–5 days estrous cycle, fertility, and fecundity), but not in rats with declining reproductive status (i.e., those that were acyclic and/or in constant diestrus or estrus, became pregnant on fewer than 60% of the occasions that they were mated, and had litters with fewer than 10 pups; Frye et al., 2010). It is possible that the use of an Ovx model, resulting in a profile of abrupt and declined reproductive status, in the present study contributed to the diminished anxiolytic effects with CEE treatment in some measures of anxiety evaluated herein. Nevertheless, these results suggest that the reproductive status of the animals could be an important determinant in modulating the estrogen regulation of anxiety.

We also found that E2 and, to a lesser extent, CEE treatments had antidepressant-like effects, as measured by decreased immobility in the FST. Although previous studies have shown antidepressant-like effects of E2 (Estrada-Camarena et al., 2003; Walf et al., 2004), this study is the first to our knowledge to demonstrate that, like E2, CEE also has some antidepressant properties. These findings in the rodent model are consistent with clinical evidence demonstrating the beneficial effects of CEE- or E2- containing HT on mood (Zweifel and O'Brien, 1997; Gleason et al., 2015). However, perhaps not unexpectedly, there are numerous parameters impacting the antidepressant effects of estrogen therapy, including menopausal status and a previous history of depression. Rubinow and colleagues reported in a systematic review of 24 randomized, placebo controlled trials, that the antidepressant efficacy of E2 was supported in perimenopausal depressed women, but not postmenopausal women or non-depressed women (Rubinow et al., 2015). Thus, further studies to reveal the specific factors and underlying mechanisms influencing the antidepressant effects of estrogens are crucial to our understanding of the nuanced effects of HT on mood.

### CEE and E2 treatments differentially increased DRN TpH2 mrna in a region-specific manner

To begin to elucidate the underlying mechanisms of estrogens on the evaluated behaviors, we examined TpH2 mRNA expression levels with a particular focus on the subregions of the DRN. It is well established that estrogen stimulates brain 5-HT activity (see Rubinow et al., 1998 for review); in particular, E2 increased TpH2 mRNA in select subregions of the DRN (Hiroi et al., 2006), and these increases in the particular subregions were critical for the anxiolytic effects of E2 (Hiroi et al., 2006, 2011). The subregions of the DRN enable distinct responses to diverse stimuli, as these subregions are recognized to have distinct inputs from, and projections to, diverse forebrain regions involved in the regulation of different aspects of behaviors (Imai et al., 1986; Vertes, 1991; Lowry, 2002). In addition to the distinct neurocircuitry, cells in the DRN subregions have unique morphology and phenotypes (Lowry, 2002). Of relevance, the two major types of estrogen receptors, ERα and ERβ, have differential expression patterns in the DRN subregions (Shughrue et al., 1997; Alves et al., 1998; Lu et al., 2001; Liu et al., 2002; Lindberg et al., 2003; Matthews and Gustafsson, 2003), enabling subregion-specific responses to different types of estrogens.

Given this subregion specificity of ER distribution, it is not surprising that we found region-specific effects of CEE vs. E2 on DRN TpH2 expression. We report here that E2 increased TpH2 mRNA in the mid and caudal, but not the rostral, subregions of the DRN, corroborating previous reports (Hiroi et al., 2006; Donner and Handa, 2009). In contrast, CEE increased TpH2 mRNA in the rostral and caudal, but not the mid, subregions of the DRN. The distinct expression patterns of ERα and ERβ in the DRN subregions may underlie a potential direct mechanism for the differential patterns of CEE- vs. E2- induced upregulation of TpH2 mRNA. Within the DRN, ERα are expressed in the non-serotonergic neurons preferentially in the rostral DRN (Alves et al., 1998). In contrast, ERβ are expressed in the serotonergic neurons of the mid and caudal DRN (Shughrue et al., 1997; Lu et al., 2001). The primary estrogen component of CEE is estrone, which has a unique pattern of ER binding affinity compared to that of E2 (Sitruk-Ware, 2002; Kuhl, 2005). Thus, further investigations to elucidate the role of region-specific ER expression in mediating estrogen effects on TpH2 expression may shed light on underlying mechanisms.

### Region-specific associations between TpH2 mRNA and distinct behaviors

In order to assess the relationships between TpH2 gene expression in the DRN and cognitive, anxiety-like, and depressive-like behaviors, we correlated the level of TpH2 mRNA in each subregion of the DRN with the evaluated behaviors. We found select associations between TpH2 mRNA and behavior, depending on the subregion and the type of behavior. A previous study showed that higher levels of TpH2 mRNA in the caudal DRN were associated with less anxiety-like behavior in the OFT (Hiroi et al., 2006); the current study also found this association. We demonstrated here that animals with higher TpH2 expression in the caudal DRN tended to exhibit decreased anxiety-like behaviors in two rodent tests of anxiety, the OFT and the EPM, suggesting that higher levels of TpH2 expression in the caudal DRN are associated with an anxiolytic profile. The importance of the caudal DRN in mediating the anxiolytic effects of E2 has also been highlighted in a previous study, as the anxiolytic effects of E2 were blocked or mimicked by a targeted TpH2 knockdown or overexpression, respectively, in the caudal DRN (Hiroi et al., 2011).

Here, we also report significant associations between depressive-like behaviors and TpH2 mRNA in select subregions of the DRN. We found that TpH2 mRNA selectively in the rostral and caudal, but not the mid, DRN was negatively correlated with the time spent immobile in the FST; thus, animals with higher TpH2 mRNA in these subregions of the DRN tended to exhibit reduced depressive-like behaviors. The neurons from the rostral DRN preferentially project to forebrain regions, such as neocortex, striatum, and substantia nigra, and are thought to be important for behavioral arousal after stress. On the contrary, the caudal DRN neurons preferentially project to limbic regions, such as ventral hippocampus, lateral septum, and locus coeruleus, and are important for the regulation of emotional coping (Vertes, 1991; Vertes et al., 1999; Lowry, 2002). Therefore, the recruitment of the rostral and caudal subregions of the DRN may help modulate important aspects of depressive-like behaviors, such as stress-response and coping strategies, required to produce an appropriate response to a stressful environment.

Cognitive behaviors also had region-selective associations with TpH2 in the current study. Indeed, we showed here that TpH2 mRNA selectively in the rostral, but not mid or caudal, DRN was negatively correlated with total errors made on the working memory trial (trial 2) of the DMS cognitive task, indicating that animals with higher TpH2 mRNA in the rostral DRN tended to exhibit better working memory performance. Compared to the caudal DRN, the rostral DRN projects more densely to virtually all neocortical regions (Vertes, 2004), which are regarded as the most recently evolved brain regions and are thought to be important for processing higher cognitive functions in mammals, such as spatial reasoning. Specifically, the frontal cortex may be an important region regulating spatial abilities in rodents and humans (Poucet, 1990; Poucet and Herrmann, 1990; Miotto et al., 1996; Bimonte-Nelson et al., 2003a). Therefore, activation of 5-HT activity from the rostral DRN may modulate cognitive performance in the DMS task via altering the cortical activity in regions such as the frontal cortex. It is noteworthy that we found that CEE, but not E2, increased TpH2 mRNA in the rostral subregions of the DRN, suggesting that the CEE-induced enhancement of DMS performance may be, in part, mediated by this increase in rostral DRN TpH2. However, the associations between the behaviors and TpH2 reported in the current study are correlational, and further studies manipulating these variables are required to confirm these assertions.

### Clinical implications

The present findings are in agreement with the human literature suggesting an intricate relationship between estrogen and the brain serotonergic system, and indicating that this interaction plays an important role in regulating behavior. Decreased 5-HT activity is associated with menopause (Halbreich et al., 1995), as well as cognitive and mood disorders (Sun et al., 2004; Garcia-Alloza et al., 2005; You et al., 2005; Mowla et al., 2007; Noristani et al., 2011; Waider et al., 2011). The beneficial cognitive and mood effects of estrogen-containing HT and agents that enhance serotonergic neurotransmission, such as selective serotonin reuptake inhibitors (SSRI), have been demonstrated (Campbell and Whitehead, 1977; Best et al., 1992; Ohkura et al., 1995; Arpels, 1996; Gregoire et al., 1996; Gleason et al., 2015). These studies support the hypothesis that estrogens ameliorate the symptoms of cognitive and mood disorders by, in part, restoring brain 5-HT activity. The present study further highlights the importance of regional specificity as a parameter to uncover the relationships amongst hormones, the serotonergic system, and behavior. Specifically, we found that CEE and E2 increased TpH2 mRNA in distinct subregions of the DRN, and that these increases in TpH2 were associated with improved cognitive performance, decreased anxiety-like behaviors, and decreased depressive-like behaviors, in a region-specific manner. This region specificity is likely key to further unravel the complex relationships between hormones, the brain serotonergic system, and regulation of distinct aspects of behaviors.

## Conclusion

In summary, the present study found that estrogen treatment had beneficial effects on cognitive, anxiety-like, and depressive-like behaviors, corroborating clinical reports; however, the effects depended on specific parameters. As CEE is becoming less popular and bioidentical hormones, such as E2, are increasingly gaining popularity and are prescribed more frequently now in the post-WHI era, the findings reported here provide a timely analysis of the differential effectiveness of CEE vs. E2. Although both CEE and E2 exerted beneficial effects, the efficacy of the treatments depended on the distinct behavior. Moreover, whether the beneficial cognitive effects of estrogens were observed was impacted by task difficulty. More robust protective effects were found when animals were challenged with a more demanding task. Compared to CEE, E2 generally had more robust anxiolytic and antidepressant effects. The hormone-specific effects on anxiety- and depressive- like behaviors further underscore the assertion that the beneficial effects of these hormones depend on the demands of the task; for example, the FST may induce a higher stress response compared to the OFT and EPM, and this could impact the efficacy of estrogens. These behavioral effects of CEE and E2 were accompanied by a differential impact on the DRN 5-HT system depending on the subregion, highlighting the importance of evaluating these subregions to understand the intricate relationships between 5-HT activity and different types of behavior. Indeed, we demonstrated that CEE and E2 increased TpH2 gene expression with remarkable region specificity. We found that E2 increased TpH2 mRNA in the mid and caudal DRN, while CEE increased TpH2 mRNA in the rostral and caudal DRN, suggesting a hormone- and region- specific enhancement of the capacity to make more 5-HT in these regions, thereby potentially increasing the serotonergic output to the select forebrain projection areas. These region-selective increases in TpH2 may have important regulatory functions, as we also found that the associations between each behavior and TpH2 depended on the distinct DRN subregion. Deciphering serotonergic involvement in the regulation of these behaviors is critical, as it may provide non-hormonal options to treat adverse symptoms of menopause. Non-hormonal options, such as the recently FDA-approved use of the SSRI paroxetine for vasomotor symptoms in menopausal women, present alternatives for women who have contraindications for the use of HT. Therefore, further studies to detail the specific serotonergic neurocircuitry involved in cognition vs. anxiety vs. mood, and how estrogens modulate these relationships, may lead to novel, non-hormonal avenues of tailored treatment for individuals suffering from cognitive and affective disorders.

## Author contributions

HBN and RH contributed to the conception or design of the work, the acquisition, analysis, and interpretation of data for the work; drafting the work; revising it critically for important intellectual content; final approval of the version to be published; agreement to be accountable for all aspects of the work in ensuring that questions related to the accuracy or integrity of any part of the work are appropriately investigated and resolved. GW, SK, SM, JT, LH, CL, PM, AJ contributed to the acquisition, analysis, and interpretation of data for the work; revising the draft critically for important intellectual content; final approval of the version to be published; agreement to be accountable for all aspects of the work in ensuring that questions related to the accuracy or integrity of any part of the work are appropriately investigated and resolved.

## Funding

This work was supported by grants awarded to RH from the National Institute of Mental Health (F32-MH093145), and by grants awarded to HBN from the National Institute on Aging (R01-AG02084), the state of Arizona, ADHS, and the Arizona Alzheimer's Disease Core Center.

### Conflict of interest statement

The authors declare that the research was conducted in the absence of any commercial or financial relationships that could be construed as a potential conflict of interest.
